# Seasonal Distribution and Genotyping of Antibiotic Resistant Strains of *Listeria* *Innocua* Isolated from A River Basin Categorized by ERIC-PCR

**DOI:** 10.3390/ijerph15071559

**Published:** 2018-07-23

**Authors:** Hsin-Chi Tsai, Ming-Yuan Chou, Cheng-Chun Wu, Min-Tao Wan, Yi-Jie Kuo, Jung-Sheng Chen, Tung-Yi Huang, Bing-Mu Hsu

**Affiliations:** 1 Department of Psychiatry, School of Medicine, Tzu Chi University, Hualien 970, Taiwan; cssbmw45@gmail.com; 2 Department of Psychiatry, Tzu-Chi General Hospital, Hualien 970, Taiwan; 3 Department of Internal Medicine, Cheng Hsin General Hospital, Taipei 112, Taiwan; colin73915@hotmail.com; 4 Department of Orthopedic Surgery, Wan Fang Hospital, Taipei Medical University, Taipei 116, Taiwan; 99231@w.tmu.edu.tw (C.-C.W.); sakiochen@msn.com (Y.-J.K.); 5 Biodiversity Research Center, Academia Sinica, Taipei 115, Taiwan; mintao@hotmail.com; 6 EcoHealth Microbiology Laboratory, WanYu Co., Ltd., Chiayi 600, Taiwan; 7 Department of Earth and Environmental Sciences, National Chung Cheng University, Chiayi 621, Taiwan; nicky071214@gmail.com (J.-S.C.); tyhuang27@gmail.com (T.-Y.H.); 8 Center for Innovative on Aging Society (CIRAS), National Chung Cheng University, Chiayi 621, Taiwan

**Keywords:** *Listeria innocua*, river basin, ERIC-PCR, diversity, antimicrobial, resistance

## Abstract

*Listeria innocua* retains many conserved homologous domains with *Listeria monocytogenes*, which is a food-borne and water-borne diarrhea-causing bacterium. Studies of antimicrobial resistance in *L. innocua* showed that this microbe is more prone to acquire resistance than other bacteria in the genus *Listeria.* However, little is known about the seasonal population distribution and antimicrobial resistance patterns of *L. innocua* in natural water environments. The aims of the study were: (1) to investigate the occurrence of *L. innocua* isolates in a subtropical watershed and reconstruct the population structure and (2) to analyze the antibacterial resistance patterns of the identified *L. innocua* isolates according to ERIC type. A total of 288 water samples was collected from the Puzi River basin (23°28’ N, 120°13’ E) between March 2014 and March 2015, and 36 *L. innocua* isolates were recovered from 15 positive water samples. With regard to seasonal variation, *L. innocua* was only detected in the spring and summer. Eighteen enterobacterial repetitive intergenic consensus (ERIC)-PCR types were identified, and two genogroups with four subgroups were reconstructed in a minimum spanning tree. Isolates from different sampling areas that were located near each other were genetically different. All *L. innocua* isolates (including 41.7% of the multidrug-resistant (MDR) isolates) were resistant to oxacillin and showed high minimum inhibitory concentrations of tetracycline. These findings demonstrate the seasonal variations and differing geographical distributions of *L. innocua* in this subtropical water environment, as well as the existence of strong population structures and MDR and antimicrobial resistance patterns. Phylogenetic analysis based on ERIC-type showed that the Cluster A isolates were resistant to more antibiotics, and two types, ERIC8 and ERIC15 were multidrug resistant. The more commonly detected types, such as ERIC1 and ERIC12, were also more likely to be resistant to two or more antibiotics. Close monitoring of drug resistance in environmental *L. innocua* is warranted due to its potential for transferring antimicrobial resistance determinants to pathogenic *Listeria.*

## 1. Introduction

*Listeria* spp. are important Gram-positive, non-spore forming, motile, facultative anaerobic microorganisms [1]. The genus *Listeria* comprises eight species, *Listeria monocytogenes*, *Listeria ivanovii*, *Listeria seeligeri*, *Listeria innocua*, *Listeria welshimeri*, *Listeria grayi*, *Listeria marthii*, and *Listeria rocourtiae* [2]. *L. monocytogenes* is one of the most important foodborne pathogens, as it can cause listeriosis in both humans and animals, whereas *L. ivanovii* is only pathogenic to animals [3]. In contrast to the major public health concern associated with *L. monocytogenes*, *L. innocua* has received relatively little attention as it is generally considered to be non-pathogenic [4]. However, a hemolytic *L. innocua* strain with *L. monocytogenes* pathogenicity island 1 genes and a human case of *L. innocua* meningitis in humans have been reported [5]. In addition, drug-resistance assays of *Listeria* have shown more antimicrobial resistance in *L. innocua* than in other *Listeria* species [6]. Because the genomes of *L. innocua* and *L. monocytogenes* have extensive homology, there is the potential for transfer of resistance genes. 

*Listeria* spp. are widespread in land and water environments [7]. Numerous studies have shown that the pathogen *L. monocytogenes* is commonly distributed in various environments, including surface water, sewage, wastewater, soil, agricultural ecosystems, and domestic environments, and that this species is particularly persistent in water environments [7,8,9,10]. A study of environmental *Listeria* spp. reported that *L. seeligeri* and *L. welshimeri* were significantly associated with natural environments, while *L. innocua* and *L. monocytogenes* were significantly associated with urban environments [10]. Like *L. monocytogenes*, *L. innocua* is able to survive under harsh conditions (e.g., high pH, high and low temperatures, and high salt concentrations) [5] and has been frequently isolated from soil, sewage, and surface water [11]. Due to the perfect synteny between the *L. innocua* and *L. monocytogenes* genomes, *L. innocua* is often used as an indicator of how widespread *Listeria* contamination is at processing facilities [6,12]. In addition, studies indicated that since *L. innocua* and *L. monocytogenes* possess numerous homologous sequences, coexistence of the two strains can lead to conjugative transposition of resistance genes, presenting a threat to public health [6,13,14]. 

Current knowledge of the seasonal distribution of *L. innocua* in subtropical water environments is poor, and little is known about the population structure of *L. innocua* in subtropical river basins. In addition, understanding the antimicrobial resistance profiles of the *L. innocua* strains in river basins will provide information regarding the antibiotic contamination in rivers. Therefore, the objectives of this study were: (i) to conduct a systematic survey of the presence and seasonal variation of *L. innocua* in a typical natural river basin in a subtropical area (Puzi River basin, Taiwan) and (ii) to reconstruct the population structure of *L. innocua* in this subtropical river basin via enterobacterial repetitive intergenic consensus-polymerase chain reaction (ERIC-PCR) and (iii) to characterize the antibiograms.

## 2. Materials and Methods

### 2.1. Sampling Sites and Surface Water Sampling

**Figure 1:**
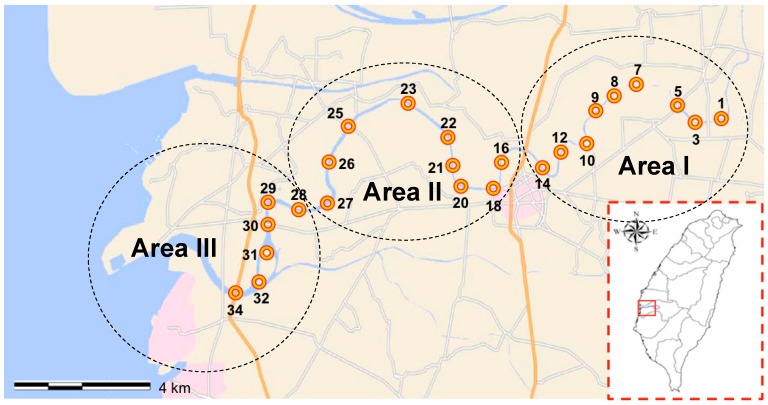
Location of water sampling sites include this study on Puzi River basin, Taiwan.

The study areas were located within the Puzi River basin in Chia-Yi county, Taiwan (23°28′ N, 120°13′ E), which lies within a classical subtropical monsoon climate region located near the Tropic of Cancer (23°26′ N). The Puzi River basin has a total length of 75.87 km, with a mean river flow rate 16 m^3^/s, and a total area of 426.6 km^2^, with roughly nine tributaries. The Puzi River basin is an important water source for local agricultural irrigation, fish farming, herding and animal husbandry, public water, recreational water activities, and various industries in western Taiwan, and it is moderately polluted [15]. Water was sampled within an approximately 140 km^2^ area of the Puzi River. The sampling site was divided into the three sampling areas (Figure 1) as described previously [15]. Seasons were defined by the Taiwan Weather Bureau as follows: spring, March to May; summer, June to August; autumn, September to November; and winter, December to February. A total of 288 water samples were collected, concentrated, and then processed for *L. innocua* isolation and for molecular analyses and antimicrobial susceptibility analysis. The temperature and pH of the samples ranged from 18.9 °C to 32.6 °C and 7 to 8.4, respectively. Samples (1 L) were taken from the water surface at each of the selected three areas between March 2014 and March 2015. The samples were stored at 4 °C, and DNA was extracted within 8 h.

### 2.2. Enrichment and Isolation of L. innocua from Water Samples

*Listeria* spp. enrichment was initiated within 24 h of water sampling as described below. The water samples were processed according to the method of Lyautey et al., with some modifications [8]. Briefly, 1 L of water was filtered through a 0.45 μm pore size, 47 mm cellulose acetate filter (Pall Gelman GN-6; VWR International, Mississauga, ON, Canada), and the filters were aseptically transferred to 10 mL of half-Fraser broth (HFB; Difco, Sparks, MD, USA) and incubated for 24 h at 30 °C. After 24 h, 100 μL of this primary enrichment broth was transferred to 10 mL of HFB and incubated for 48 h at 37 °C. The HFB from the secondary enrichment was streaked onto a CHROMagar™ Listeria (CHROMagar™ Microbiology, Paris, France) plate and incubated for 24 h at 37 °C. Up to five different suspected *Listeria* colonies (with blue morphology) were chosen. These colonies were individually inoculated in 5 mL of HFB and incubated for 24 h at 37 °C, and then a loopful of the specimen was streaked to polymyxin-acriflavin-lithium chloride-ceftazidime-aesculin-mannitol (PALCAM) agar (Difco) for 24 h at 37 °C. Gray-green colonies were suspected to be *Listeria* spp. All isolates were stored at −80 °C until analysis. The genomic DNA of suspected *Listeria* spp. was purified using the MagPurix Bacteria DNA Extraction Kit (ZP02006; Zinexts Life Science Corporation, New Taipei City, Taiwan) according to the manufacturer’s instructions. The identity of suspected *L. innocua* colonies was confirmed by PCR amplification of the *prs* and *iap* genes of *Listeria* spp. and *L. innocua*, respectively [16,17].

### 2.3. ERIC-PCR for L. innocua Isolates

ERIC-PCR was performed as described by Rivera et al., with some modifications [18]. Primers ERIC-1 (5′-ATG TAA GCT CCT GGG GAT TCA C-3′) and ERIC-2 (5′-AAG TAA GTG ACT GGG GTG AGC G-3′) were used. Reactions mixtures (25 μL) contained 1 μM each primer, 100 ng of genomic DNA, 1.5 mM MgCl_2_, 0.2 mM each deoxynucleoside triphosphate (dATP, dTTP, dCTP, and dGTP; Promega, Fitchburg, WI, USA), and 1 U of *Taq* DNA polymerase (Promega). The reaction mixture was denatured at 95 °C for 5 min. Then, the PCR amplification was performed as follows: 35 cycles of denaturation at 92 °C for 45 s, annealing at 52 °C for 1 min, and extension at 70 °C for 10 min, with a final extension at 70 °C for 20 min. Amplification products were resolved in 1.8% agarose at 60 V for 6 h, stained with ethidium bromide, and analyzed as described. The 100 bp DNA Ladder (Promega) was used as a molecular size marker.

### 2.4. Population Structure and Minimum Spanning Tree Reconstruction

A minimum-spanning tree (MST) and the geographical information for *L. innocua* haplotypes were reconstructed using Phyloviz 1.0 software (http://goeburst.phyloviz.net/) [19] to infer the population structure and geographical distribution among the isolates.

### 2.5. Antimicrobial Susceptibility and Minimum Inhibitory Concentration

The resistance breakpoints of the *L. innocua* isolates to ciprofloxacin (≥2 μg/mL), clindamycin (≥2 μg/mL), oxacillin (≥2 μg/mL), rifampin (≥ 4 μg/mL), tetracycline (≥16 μg/mL), and trimethoprim/sulfamethoxazole (≥4 μg/mL) was determined using the agar dilution method on Mueller-Hinton agar according to Clinical and Laboratory Standards Institute (CLSI) protocol M45-A [20]. Multidrug resistance (MDR) was defined as concurrent resistance to two or more different antimicrobial mechanisms [21]. The minimal inhibitory concentration (MIC) of the *L. innocua* isolates was determined using the broth micro-dilution method according to CLSI guideline M31-A3 [22].

### 2.6. Statistical Analysis

STATISTICA 8.0 (StatSoft, Inc., Tulsa, OK, USA) was used for statistical analyses. The distribution of the ERIC-PCR genotypes among the population and the antimicrobial susceptibility components in the *L. innocua* isolates were assessed using the chi-square test for homogeneity of proportions. A *P* value less than 0.05 was considered statistically significant.

## 3. Results

### 3.1. The Presence and Seasonal Distribution of L. innocua in the Puzi River Basin

A total of 288 surface water samples were analyzed for the presence of *L. innocua*, and the results are shown in Table 1. 

Among these samples, 15 were positive, and 36 *L. innocua* strains were isolated. The samples from area I had the highest detection rate (11.5%, 11/96), followed by area II at 4.2% (4/96). No *L. innocua* was detected in the samples from area III (0/96). The sampling site with the highest detection rate was PR14 at 25% (3/12). *L. innocua* was only detected during the spring and summer. The highest seasonal occurrence was during the spring in area I (41.7%, 10/24), followed by during the spring in area II (12.5%, 3/24) and during the summer in areas I and II (4.2%, 1/24, for both).

### 3.2. ERIC-PCR and Minimum Spanning Tree Analysis

**Figure 2:**
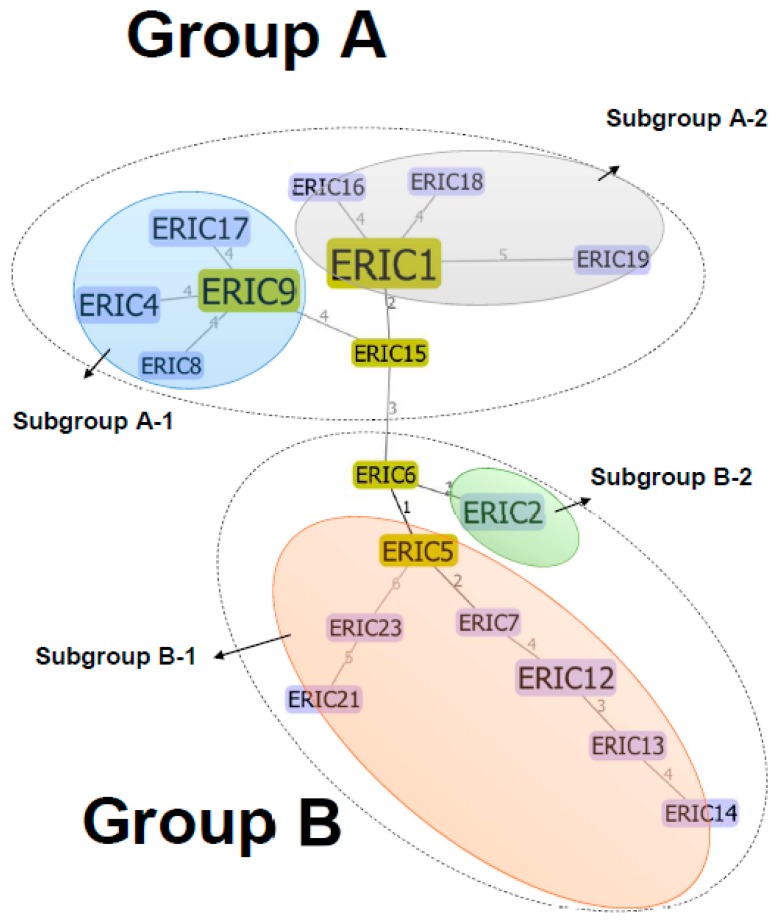
Minimum spanning tree based on ERIC analysis of 36 *L. innocua* isolates.

All 36 isolates were examined and characterized by ERIC-PCR. A visual comparison of the banding patterns revealed 18 distinct ERIC profiles, with DNA fragments ranging from 100 to 3000 bp (data not shown). The predominant ERIC type was ERIC 1 (25%, 9/36), followed by ERIC 2, ERIC 4, ERIC 12, and ERIC 17 (8.3%, 3/36); ERIC 5 and ERIC 9 (5.6%, 2/36); and ERIC 6-8, ERIC 13-16, ERIC 18, ERIC19, ERIC21, and ERIC 23 (2.8%; 1/36). The minimum spanning tree showed that the 18 ERIC types of the *L. innocua* isolates from the Puzi River basin were further divided into two major groups and that each of these groups contained two subgroups (Figure 2). Group A included 17 strains from area I and five strains from area II, and cluster B included 12 strains from area I and two strains from area II.

### 3.3. Geographical Distribution of L. innocua in the Puzi River Basin

**Figure 3:**
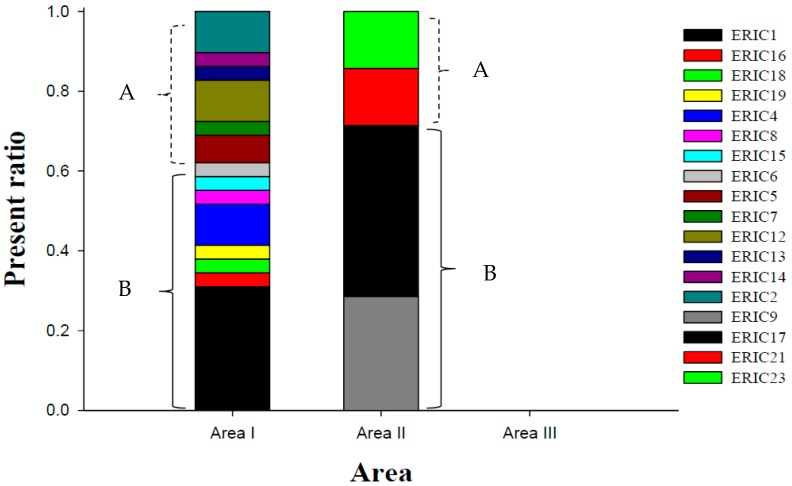
Geographical distribution of the *L. innocua* ERIC types in the three sampling areas. A is group A, and B is group B as determined by the minimum spanning tree analysis shown in Figure 2.

The geographical distribution of the ERIC types is shown in Figure 3. There was a significant difference in the numbers of the different ERIC types between areas I and II (*p* < 0.05). Areas I and II contained 14 and 4 ERIC types, respectively. The predominant ERIC type in area I was ERIC 1 (31%, 9/29), followed by ERIC 2 (10.3%, 3/29), ERIC 4 (10.3%, 3/29), and ERIC 12 (10.3%, 3/29); these four ERIC types accounted for 62.1% (18/29) of all the isolates in area I. In area II, the predominant ERIC type was ERIC 17 (42.9%, 3/7), followed by ERIC 9 (28.6%, 2/7); these 2 ERIC types accounted for 71.4% (5/9) of the isolates in area II (Figure 3). None of the ERIC types were present in both areas I and II.

### 3.4. Antimicrobial Susceptibility and Minimum Inhibitory Concentration (MIC)

**Figure 4:**
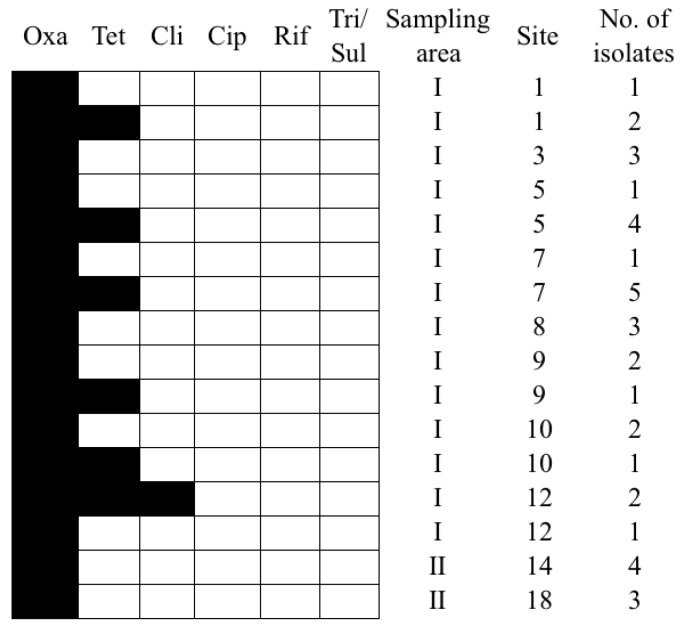
Antibiograms and the source of *L. innocua* isolates recovered from the Puzi River basin, Taiwan. Black indicates resistant, and white indicates susceptible. Oxa: Oxacillin; Cli: Clindamycin; Tet: Tetracycline; Rif: Rifampin; Cip: Ciprofloxacin; Tri/Sul: Trimethoprim/Sulfamethoxazole.

All isolates were resistant to oxacillin and susceptible to ciprofloxacin and rifampin, and 15 of the *L. innocua* isolates were MDR (41.7%, 15/36). More than 40% of the isolates were resistant to tetracycline, and <10% were resistant to clindamycin and trimethoprim/sulfamethoxazole. The antibiograms of the *L. innocua* isolates from area I were significantly different from those of the isolates from area II (*p* < 0.05; Figure 4). The predominant resistance patterns among the area I isolates were Oxa only (48.3%, 14/29) and Oxa-Tet (44.8%, 13/29); these two resistance patterns accounted for 93.1% (27/29) of all area I isolates (Figure 4). In contrast, the isolates from area II were only resistant to oxacillin (100%, 7/7; Figure 4). Among the area I isolates, 75.9% had a MIC of tetracycline ≥2 μg/mL, and 69% had a MIC of oxacillin ≥4 μg/mL. There was a significant difference in the MIC of tetracycline between the isolates from areas I and II (*p* < 0.05).

### 3.5. Comparison of Antimicrobial Susceptibility According to ERIC-Type

We summarized the ERIC-types and antimicrobial susceptibility of the isolates (Table 2). Cluster A presented more resistance to distinct combinations of antibiotics, excluding oxacillin. The most common resistance pattern among ERIC8 and ERIC15 isolates was oxacillin + clindamycin + tetracycline + ciprofloxacin + trimethoprim/sulfamethoxazole. ERIC1 strains were resistant to oxacillin + tetracycline, and one strain was highly resistant to oxacillin (4 µg/mL). The ERIC8 strain presented higher antibiotic susceptibility to clindamycin (8 µg/mL) and ciprofloxacin (4 µg/mL). All of the isolates that were resistant to ciprofloxacin with higher antibiotic susceptibility (4 µg/mL).

## 4. Discussion

This study shows the seasonal variation, geographical distribution, population structure, and antimicrobial susceptibility of *L. innocua* in a subtropical river basin. First, we determined the seasonal and geographical variations of *L. innocua*. Second, we demonstrated the high genetic diversity and highly diverse population structures of *L. innocua* in the Puzi River basin of Taiwan. Third, we showed the multidrug-resistance patterns among the *L. innocua* isolates. Finally, we showed that the *L. innocua* isolates from each sampling area of the basin had unique resistance profiles.

The overall detection rate of *L. innocua* in the water samples from the Puzi River basin was 5.2% (15/288), which is much lower than the rate previously reported in urban and rural agricultural watersheds of Canada (32.8%) [7] and slightly higher than the rate reported in urban and rural environments in the USA (2.3%) [10]. These results demonstrate the variability in the occurrence of *L. innocua* in different countries and geographical areas. Previous studies have documented seasonal effects on the prevalence of various *Listeria* spp. in natural environments [10,23,24]. Consistent with these earlier reports, we also observed a clear seasonal trend in the distribution of *L. innocua* in a subtropical water environment.

In the present study, the highest prevalence was observed in the spring (March–May), which differs from that reported by Sauders et al., who observed the highest prevalence in the summer, and by Stea et al., who observed the highest prevalence of *L. monocytogenes* in the cooler and cold months of September–February. These differences may be due to variations in the ecological preferences and adaptations of different *Listeria* spp., even though *L. innocua* and *L. monocytogenes* are genetically similar.

The use of ERIC-PCR typing has been reported to be useful for epidemiological analyses of *Listeria* spp., including *L. monocytogenes* [25,26,27]. To our knowledge, ERIC-PCR typing has not been used to construct a population structure of highly clonal *L. innocua* isolated from environmental water samples. In the present study, 18 ERIC types were identified (Figure 2). These findings suggest that the ERIC region could reflect the diverse genetic background of *L. innocua* and may be a useful molecular marker for tracking the source of *L. innocua* isolates and reconstructing the initial structure of *L. innocua* populations found in different geographical areas.

A previous study demonstrated that *L. monocytogenes* populations can be divided into several distinct subgroups, even those that belong to the same lineage [28]. In addition, different countries and regions may have different populations of microbes, such *Staphylococcus aureus* [29]. In this study, integration of a minimum spanning tree (MST) based on ERIC-PCR typing showed high genetic diversity among the *L. innocua* isolates, with unique population structures in areas I and II (Figure 2), and this particular heterogeneity feature was observed here for the first time. The MST showed a highly variable number of ERIC types for two distinct groups, and two possible ancestral types, ERIC 6 and ERIC 15, were suggested (Figure 2). Interestingly, we found that the predominant types of *L. innocua* in areas I and II were different (ERIC 1 in area I and ERIC 17 in area II; Figure 3). This is likely due to strict ecological niches or the effects of environmental variables, even though these two sampling areas are close to each other. In addition, there were four unique subgroups originating from groups A (subgroups A1 and A2) and B (subgroups B1 and B2). We believe that these two groups have persisted and evolved in the Puzi River basin, although the past population dynamics are unknown.

More than 40% of the *L. innocua* isolates identified in this study were MDR, and the isolates from areas I and II had significantly different antibiograms. The differences in the resistance profiles of the isolates from these two areas may be due to their differing geographical origins. We also noted that there is a higher rate of resistance to tetracycline, which is on the list of approved feed additives in Taiwan, along with gentamicin, tetracycline, trimethoprim, and sulfonamides [29]. The observed resistance and high MIC might be residual effects of low-dose tetracycline use and pharmaceutical contamination due to medication use by humans and/or from feed additives used in the livestock industry [29,30]. This contamination may provide selective pressure in the water environments and may also affect the antibacterial responses of normal flora, such that resistance may be transferred to the community via recreational water exposure. Since *L. innocua* easily acquires resistance, it may act as an early warning sign of antimicrobial pollution. Highly prevalent genotypes and strains with broad host range may have greater dissemination capability as well as a higher chance of interacting with antibiotic-resistant microbes and evolving into resistant phenotypes, especially in an environment containing residual antibiotics, especially our study area, which has been identified as a polluted river basin by the government.

## 5. Conclusions

In this study, the distribution and antimicrobial susceptibility of *L. innocua* isolates in the Puzi River watershed were analyzed. The highest prevalence of *L. innocua* was found in area I, which also had the highest percentage of antibiotic-resistant strains. The prevalence of *L. innocua* and antibiotic-resistant strains was significantly decreased in area II, and no strains were isolated from area III. Since area I was located in a primary area of animal husbandry, the occurrence of antibiotic-resistant bacteria may be associated with antimicrobial use. Although *L. innocua* is non-pathogenic, the study of MDR and the MICs of various antimicrobial agents provided us with references for the surveillance of antibiotic usage and the evolution of antibiotic-resistant bacteria, which will be useful for the control of antibiotic-resistant bacteria for public health purposes. To our knowledge, this is the first study to investigate the seasonal distribution of *L. innocua* in a typical subtropical watershed over a one-year period. The presence of MDR *L. innocua* in this river basin implies that the river has antibiotic contamination, and the horizontal gene transfer ability of *L. innocua* poses a serious threat to public health.

## Figures and Tables

**Table 1:** *L. innocua* results for three sampling areas of 4 seasonal.

Area	Seasonal	Number of Samples	*L. innocua* Detection Rate	*L. innocua* Isolates (No.)
I	Spring	24	41.7% (10)	27
	Summer	24	4.2% (1)	2
	Fall	24	0	0
	Winter	24	0	0
	Total	96	11.5% (11)	29
II	Spring	24	12.5% (3)	5
	Summer	24	4.2% (1)	2
	Fall	24	0	0
	Winter	24	0	0
	Total	96	4.2% (4)	7
III	Spring	24	0	0
	Summer	24	0	0
	Fall	24	0	0
	Winter	24	0	0
	Total	96	0	0
Total		288	5.2% (15)	36

**Table 2:** Proportion of *L. innocua* isolates from a river basin in Taiwan showing resistance (%) to six antimicrobial agents by ERIC type.

Cluster	Eric-Type	Antimicrobial Agents					
		Oxacillin (2 µg/mL)	Clindamycin (2 µg/mL)	Tetracycline (16 µg/mL)	Rifampin (4 µg/mL)	Ciprofloxacin (2 µg/mL)	Trimethoprim/Sulfamethoxazole (4 µg/mL)
A-1	ERIC 4 (*n* = 3)	100% (2 µg/mL)	0	0	0	0	0
	ERIC 8 (*n =* 1)	100% (4 µg/mL)	100% (8 µg/mL)	100% (16 µg/mL)	0	100% (4 µg/mL)	100% (4 µg/mL)
	ERIC 9 (*n* = 2)	100% (2 µg/mL)	0	0	0	50% (4 µg/mL)	0
	ERIC 17 (*n* = 3)	100% (2 µg/mL)	0	0	0	0	0
A-2	ERIC 1 (*n* = 9)	100% (2 µg/mL) *	0	100% (16 µg/mL)	0	0	0
	ERIC 16 (*n* = 1)	100% (2 µg/mL)	0	0	0	100% (4 µg/mL)	0
	ERIC 18 (*n* = 1)	100% (4 µg/mL)	0	0	0	0	0
	ERIC 19 (*n* = 1)	100% (2 µg/mL)	0	100% (16 µg/mL)	0	0	0
Out-group A	ERIC 15 (*n* = 1)	100% (8 µg/mL)	100% (4 µg/mL)	100% (16 µg/mL)	0	100% (4 µg/mL)	100% (4 µg/mL)
B-1	ERIC 5 (*n* = 2)	100% (2 µg/mL)	0	0	0	0	0
	ERIC 7 (*n* = 1)	100% (2 µg/mL)	0	0	0	0	0
	ERIC 12 (*n* = 3)	100% (2 µg/mL) *	0	100% (16 µg/mL)	0	33.3% (4 µg/mL)	0
	ERIC 13 (*n* = 1)	100% (2 µg/mL)	0	0	0	0	0
	ERIC 14 (*n* = 1)	100% (8 µg/mL)	0	0	0	0	0
	ERIC 21 (*n* = 1)	100% (2 µg/mL)	0	0	0	0	0
	ERIC 23 (*n* = 1)	100% (2 µg/mL)	0	0	0	0	0
B-2	ERIC 2 (*n* = 3)	100% (2 µg/mL)	0	0	0	0	0
Out-group B	ERIC 6 (*n* = 1)	100% (2 µg/mL)	0	0	0	0	0
Total	*n* = 36	100%	5.6%	41.7%	0	13.9%	5.6%

The maximum antibiotic concentration of *L. innocua* was attached after each strain; * One isolate was resistant to 4 µg/mL oxacillin.
